# Ultra-Processed Food Intake in Children with Inflammatory Bowel Disease: A Pilot Case–Control Study

**DOI:** 10.3390/nu17223532

**Published:** 2025-11-12

**Authors:** Emese Kasznár, Dorina Bajzát, Anna Karoliny, Judit Szentannay, András Szabó, Eszter Gombos, Vivien Regián, Anikó Havasi, Erzsébet Pálfi, Katalin Eszter Müller

**Affiliations:** 1 Heim Pál National Pediatric Institute, 1089 Budapest, Hungary; 2 Centre for Translational Medicine, Semmelweis University, 1085 Budapest, Hungary; 3 Department of Dietetics and Nutrition Sciences, Semmelweis University, 1085 Budapest, Hungary; 4 Hungarian Dietetic Association, 1034 Budapest, Hungary; 5 Department of Family Care Methodology, Faculty of Health Science, Semmelweis University, 1085 Budapest, Hungary

**Keywords:** inflammatory bowel disease, ultra-processed food, diet, Crohn’s disease, ulcerative colitis

## Abstract

**Background**: The consumption of ultra-processed foods (UPFs) has increased globally, particularly in developed countries. UPFs are energy-dense and nutrient-poor, and they often contain additives that can disrupt gut flora and increase intestinal permeability. There is evidence to suggest that processed foods may contribute to the onset of IBD and also impact its progression and response to treatment. This study investigated whether children with IBD consume more UPFs than healthy controls and examined the association between UPF intake and disease activity. **Methods**: This pilot cross-sectional case–control study recruited children with IBD from the Gastroenterology Outpatient Clinic at the Heim Pál National Pediatric Institute in Budapest, Hungary, between December 2023 and February 2025. Age- and sex-matched healthy controls (HCs) were also enrolled. Dietary intake was assessed using two days of 24 h recalls. UPF intake was categorized using the NOVA system and expressed as a percentage of total daily energy intake. **Results**: A total of 47 children with IBD were matched with HCs. There was no difference in total energy intake between the two groups. Children with UC had a significantly higher intake of UPFs than HCs (MD: 10.5%, *p* = 0.02), whereas no difference was observed in children with CD after excluding oral nutritional support. No difference in UPF intake was observed between children with active or inactive disease. However, children receiving biological therapy consumed significantly fewer UPFs than those receiving other treatments (MD: 8%, *p* = 0.04). **Conclusions**: Children with IBD consume more UPFs compared to HC. The UPF intake of children with CD was not lower than healthy children despite the recommended Crohn’s Disease Exclusion Diet (CDED).

## 1. Introduction

The consumption of ultra-processed foods (UPF) has increased significantly worldwide in recent decades, especially in developed countries, where they can account for up to half of daily energy intake [1]. UPFs are typically low in nutritional value but high in energy, and industrially produced products contain a wide range of additives [2]. In recent years, an increasing number of epidemiological studies have demonstrated a link between UPF consumption and several chronic diseases, including obesity, metabolic syndrome, type 2 diabetes, cardiovascular diseases, cancers, and inflammatory bowel disease (IBD) [3,4].

The exact pathomechanism of IBD, which is becoming more prevalent worldwide, is unknown. Current knowledge suggests that the most important pathogenic factors include genetic predisposition, the immune system, gut flora, and abnormal gut mucosal function, as well as certain environmental factors [5]. Diet plays a major role among these environmental factors, as it can alter the gut flora, damage the intestinal barrier, activate inflammatory processes, and lead to energy and macronutrient over-consumption [6].

According to a recent meta-analysis, a Western diet characterized by increased consumption of processed foods and additives increases the risk of IBD [7]. Ex vivo and animal studies have also shown that food additives (e.g., emulsifiers and stabilizers) are associated with the development of dysbiosis and increased intestinal permeability [8]. Furthermore, the role of diet in pathogenesis is supported by the fact that exclusive enteral feeding is an effective induction therapy for pediatric Crohn’s disease (CD) and is currently the first-line treatment for mild to moderate cases [9].

Lamers et al. found an association between disease activity and the inflammatory potential of the diet in participants with Crohn’s disease. Those with a more pro-inflammatory diet appear to experience higher levels of disease activity [10].

This raises the question of whether processed food consumption may play a role not only in triggering the disease but also in determining its course and how it responds to therapy. Some studies have also found associations between disease activity, inflammatory markers, and diet in IBD patients.

Considering these findings, our study aimed to answer the following research questions: Do children with IBD consume more UPF than healthy children? Is there an association between UPF intake and disease activity or treatment?

## 2. Materials and Methods

### 2.1. Study Design

In this pilot cross-sectional, case–control study to assess UPF intake of children with IBD, we consecutively recruited patients from the Gastroenterology Outpatient Clinic at the Heim Pal National Pediatric Institute (Budapest, Hungary) between December 2023 and February 2025. Inclusion criteria for the IBD group were age between 12 and 18 years, disease duration, and signed informed consent. Exclusion criteria included exclusive enteral nutrition and intellectual disability. Age and sex-matched healthy controls (HC) were recruited during the same period from one primary and one secondary school in Budapest. In case of HC, the exclusion criteria were lack of signed informed consent, any underlying disease affecting diet (e.g., celiac disease), and intellectual disability. Matching was performed in a 1:1 ratio.

### 2.2. Data Collection

To assess daily energy and UPF intake, two trained dieticians (A.H., V.R.) conducted two days of 24 h dietary recalls (one weekday and one weekend) for each participant. For all children, age, sex, weight, and height were recorded. For patients with IBD, additional medical data were collected during the inclusion visit, including diagnosis, disease duration, disease localization, and phenotype (according to the Paris Classification [11]), current medications, and disease activity indices (PCDAI [12] or PUCAI [13]), clinical and laboratory parameters, and/or fecal calprotectin. These data were documented in a standardized clinical recording form using Microsoft Excel [14]. The diagnosis of IBD was made according to the Porto criteria [15], and the condition was managed in accordance with the current ESPGHAN guidelines [16,17,18]. Patients were classified as being in remission if their PUCAI or PCDAI score was below 10.

### 2.3. Assessment of Energy and UPF Intake

The dietary data obtained from the 24 h recalls were entered into Nutricomp 5.0 DietCAD Software, a Hungarian professional nutrition analysis software used to evaluate dietary intake by calculating the energy and nutrient content of foods based on standardized food composition databases. Next, we classified each food in the 24 h recalls according to NOVA classification system1 to identify ultra-processed products. To improve the accuracy of classification, two researchers (V.R, K.E.M.) independently categorized the foods. Any disagreements or uncertainties were resolved through consultation with an experienced, certified dietitian (E.P.)

### 2.4. Statistical Methods

Demographic and clinical data were summarized using descriptive statistics. For continuous variables, mean and standard deviation (SD) or minimum-maximum values were calculated, while categorical variables were presented as frequencies and percentages.

We first compared total energy intake and the percentage of energy from UPFs between HC and patients with IBD using independent two-sample *t*-tests and one-way ANOVA test with Bonferroni post hoc analysis. Additional subgroup analyses were performed within the IBD group to compare UPF intake between: (1) patients with ulcerative colitis (UC) and CD; and (2) patients in remission (PUCAI/PCDAI < 10) versus those with active disease (PUCAI/PCDAI > 10). Furthermore, we compared UPF intake between patients treated with and without biological therapies. Finally, we examined UPF intake in a subgroup of patients who required dose escalation of anti-TNF therapy or switching to another biological (escalated biological treatment group) and compared them to patients on their first biological (in Hungary, it is anti-TNF according to the current insurance protocols) and to those not receiving biologicals. Children with body mass index (BMI) z-score values below −1 were considered to be underweight, values above 1 were considered to be overweight, and values above 2 were considered to be obese [19]. Based on this, we distinguished three groups: 1. Normal weight, 2. Overweight and obese, 3. Underweight. The results were considered significant if *p* < 0.05. For statistical analyses, Microsoft Excel [14] and IBM SPSS Version 25 [20] were used.

The study was approved by the Scientific and Research Ethics Committee of the Medical Research Council on 9 May 2025 (BM/12402-1/2024).

### 2.5. Artificial Intelligence

In preparing this work, the authors utilized AI technology, specifically ChatGPT 4.0, DeepL (web version accessed June 2025, https://www.deepl.com) and Grammarly (web version accessed June 2025, https://www.grammarly.com). These tools were used for writing assistance and language translation, contributing to the efficiency and quality of the content. While AI provided valuable support, all final editing and interpretations were performed by the authors.

## 3. Results

### 3.1. Study Population

A total of 47 children with IBD completed the food diary and were matched to an HC based on age and sex. Two patients had IBD-U and were grouped with UC patients for the statistical analyses. The mean age of children with IBD was 13.2 ± 2.2 years, 42.6% of whom were female, and 51% had CD. The majority of patients (85.1%) were in remission, defined as having a disease activity index below ten. Of the 47 patients, 23 (48.9%) received biological therapy, 24 (51%) were prescribed azathioprine, and five (10.6%) were on corticosteroid therapy. The BMI z-scores indicated that 12 patients were underweight, and 11 were overweight or obese. In the HC group, five children were underweight, and six were overweight or obese. Table 1 provides a summary of the basic characteristics of the participants included in the study.

### 3.2. Energy and UPF Intake

**Figure 1:**
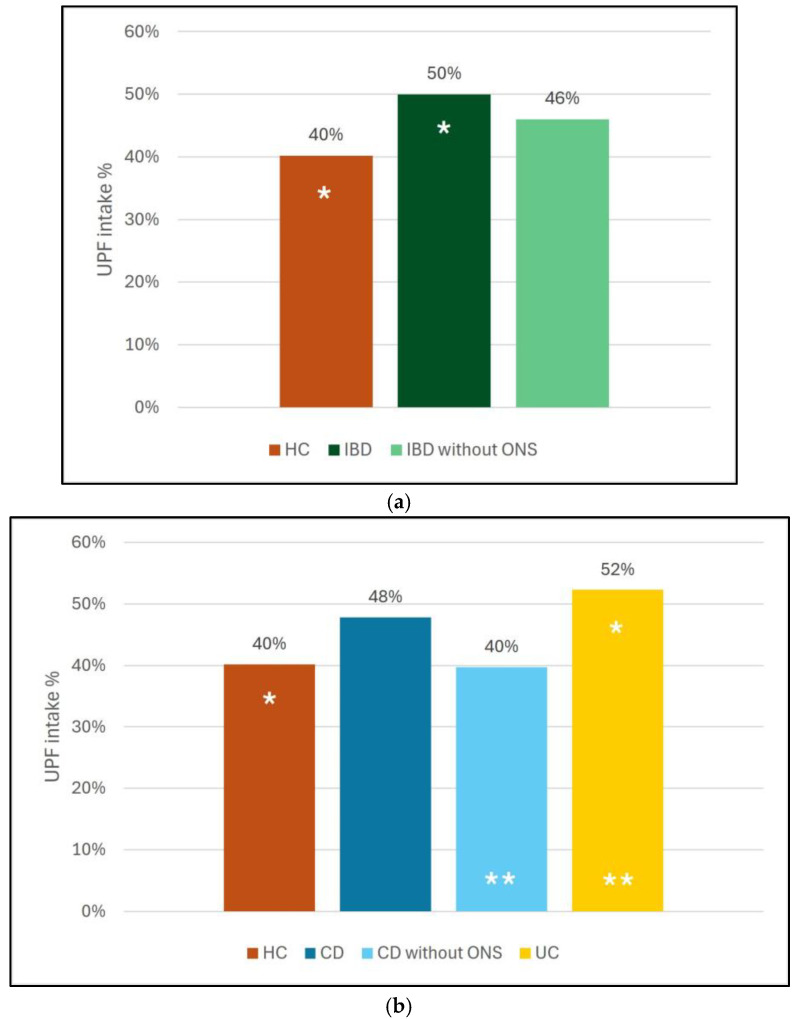
(**a**) Daily UPF intake as a percentage of total energy intake in patients with IBD and healthy controls (* mean significant difference between the groups). (**b**) Daily UPF intake as a percentage of total energy intake in patients with CD, UC, and healthy controls (* or ** mean significant difference between the groups). UPF: ultra-processed food, HC: healthy controls, IBD: inflammatory bowel disease, ONS: oral nutritional support, CD: Crohn’s disease, UC: ulcerative colitis.

The total energy intake of patients with IBD was similar to that of HC (HC: 2428 ± 995 kcal, IBD: 2764 ± 998 kcal). However, a significant increase was observed in patients with UC when compared to HC (mean difference [MD]: 740.7 kcal, *p* = 0.005). We then compared the proportion of UPF in the daily food intake between the groups. Patients with IBD consumed significantly more UPF than HC (MD: 9.9%, *p* = 0.003). (Figure 1a) When we compared the subtypes of the disease, the difference remained significant only in the case of UC (UC vs. HC MD: 10.5%, *p* = 0.02, CD vs. HC MD: 9.2%, *p* = 0.06). (Figure 1b) The UPF intake did not differ significantly between patients with UC and CD (MD: 2%, *p* = 0.29) (Figure 1b).

Some patients with CD regularly consumed oral nutritional supplements (ONS) as partial enteral nutrition. These ONS were classified as UPF in the 24 h recalls. We hypothesized that their inclusion might artificially increase the apparent intake of UPF and give a misleading impression of poor dietary choices. Therefore, we also performed the analysis excluding ONS. In this analysis, we found that UPF intake of patients with IBD did not differ significantly from HC. (MD: 5.7%, *p* = 0.84) (Figure 1a) Meanwhile, patients with UC had higher UPF intake than HC and patients with CD (UC vs. HC MD: 10.5%, *p* = 0.02, UC vs. CD MD: 12%, *p* = 0.01) (Figure 1b).

### 3.3. Disease Activity and UPF Intake

To investigate the relationship between UPF intake and disease activity, we compared children with active disease and those in remission, defined by disease activity indices. The analysis revealed no significant difference in UPF intake between the two groups (MD: 1%, *p* = 0.86) (Supplementary Figure S1). We also compared those children who were in remission for more than 1 year (n = 20) to those who had active disease based on clinical, laboratory parameters, and/or fecal calprotectin (n = 7), but again, we did not find any difference in UPF intake (MD: 5%, *p* = 0.41) (Supplementary Figure S2).

### 3.4. Need for Biologicals and UPF Intake

Twenty-three patients received biological therapy. In six cases, biological therapy was initiated due to perianal disease; in nine cases, due to clinical activity and/or steroid dependence; in two cases, due to azathioprine intolerance; and in six cases, due to unresolved calprotectin during clinical remission. Four patients required a switch from anti-TNF to a second-line biological treatment, while eight patients needed treatment escalation. First, UPF intake was compared in patients who received biological therapy and those who did not. Patients who were on biological treatment exhibited a substantial decrease in UPF intake (MD: 8%, *p* = 0.04). However, upon comparison of the UPF without ONS, the initial significance became obscured (MD: 6%, *p* = 0.22). (Supplementary Figure S3) There was no difference when we performed the analyses separately in CD and UC (CD MD: 8%, *p* = 0.24; UC MD: 11%, *p* = 0.13) (Supplementary Figure S4).

**Figure 2:**
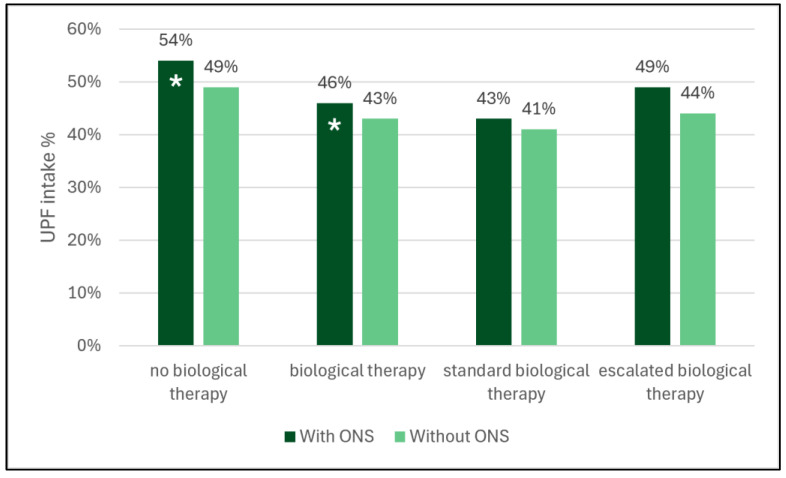
Therapy and the daily UPF intake percentage (* mean significant difference between the groups). UPF: ultra-processed food, ONS: oral nutritional support.

When we compared the escalated biological treatment group to those who received their first biological therapy (standard dose), the difference in UPF intake was non-significant (MD: 6%, *p* = 0.32). Although the comparison of treatment escalated children with those who had not received biological therapy, we saw a significant decrease (MD: 11%, *p* = 0.03) in UPF intake. Nevertheless, upon comparison of the intake after excluding ONS, the initial significance became obscured (MD: 8%, *p* = 0.18) (Supplementary Figure S5, Figure 2).

### 3.5. Energy and UPF Intake in IBD Patients Based on BMI Z-Score

Eleven patients (five with UC and six with CD) were overweight/obese based on their BMI z-scores. They had the lowest energy intake but the highest UPF intake, though the difference was not significant (Table 2).

### 3.6. Association of UPF Intake and Age, Sex, and Energy Intake

Girls with IBD had significantly higher energy intake compared to HC (MD: 473 kcal, *p* = 0.03), but their BMI z-scores did not differ significantly (MD: 0.4, *p* = 0.37). Furthermore, female patients consumed significantly more UPF (females MD: 11%, *p* = 0.03), but the difference became non-significant (MD: 6%, *p* = 0.23) after exclusion of ONS. We did not find similar differences in boys.

We also divided the population into two groups: those aged 14 years or under, and those aged 15 years and over. Nine patients were included who were aged 14 years or under, and 38 were aged 15 years and over. We observed a statistically significant increase in UPF intake in older IBD patients compared to older healthy controls (MD: 9%, *p* = 0.01), but this significance disappeared when we excluded ONS.

## 4. Discussion

This pilot cross-sectional, case–control study is the first study investigating UPF intake in children with IBD. Our findings indicate a significantly higher intake of UPFs in patients with IBD, particularly among those with UC. However, this discrepancy was influenced by the incorporation of ONS, which are frequently prescribed as part of clinical nutritional support in IBD and are technically classified as UPFs. When ONS was excluded from the analysis, the difference in UPF intake between IBD and HC was no longer statistically significant, except in the UC subgroup. Furthermore, we found no association between disease activity and disease course. Finally, our data suggest that UPF intake was somewhat lower in patients who needed biologicals and treatment escalation than in those who were in remission with immunomodulator and 5-ASA maintenance treatment.

The average UPF intake of the HC group was 40.2%, which is consistent with data found in the literature. A previous study involving the Hungarian population found that UPFs accounted for 45.8% of the average energy intake of Hungarian adult participants [21]. In Greece, in the pediatric population, this proportion was 39% [22]; in Turkey, it was 25% [23]; and in higher-income countries such as the United Kingdom and Canada, it reached 75% and 51% [24,25].

We found that UC patients had higher UPF intake than children with CD after the exclusion of ONS.

Although previous studies have examined the prevalence of enteral nutrition among patients, this was not taken into account in the assessment of UPF intake and disease characteristics [26,27]. However, if our aim is to evaluate the food choices of our patients, it is an important aspect. In our center, more than 90% of children with CD receive EEN as induction therapy and are usually advised to follow the Crohn’s Disease Exclusion Diet (CDED), though the adherence is not high. Patients with UC are usually advised to avoid UPF, but, unlike with CDED, there are no clear guidelines. Therefore, it is not surprising that the result confirms that children with CD who follow partial enteral nutrition and/or CDED have lower UPF intake than children with UC. However, their UPF intake is not lower than that of healthy children reflecting the low adherence to CDED. In adult studies, the range of UPF intake was 17–45%, though the methodology is heterogeneous (FFQ, 3-day dietary diary) [26,28,29,30]. Furthermore, it is also known that the UPF intake varies by geography [22]. Severo et al. reported that adults with IBD consumed less UPF than healthy controls [29]. Meanwhile, in another study, adults with CD had a higher intake of food additives compared to healthy controls [5]. The controversy can be explained by methodological factors, but different centers may give different dietary advice that can also contribute to the heterogeneous results in these studies. Another important factor that has an impact on the UPF intake is food insecurity, which has been described by Gold et al. Their data suggest that food insecurity may be a limiting factor in decreasing UPF intake [26].

Furthermore, this issue also raises a methodological question: should ONS be included in the calculation of UPF intake? On one hand, ONS products that are effective in inducing remission in pediatric CD are generally considered safe regarding their content of food additives [31]. On the other hand, their emulsifiers and additive content contribute to overall UPF intake and should be overlooked as these components may negatively affect the intestinal permeability, intestinal composition, and inflammation. Sarbagili-Shabata et al. excluded patients on elimination diet and/or partial enteral nutrition during the assessment of the relationship between disease activity index and UPF intake and found that the association still remained [27].

Large epidemiological studies have described an association between UPF and the incidence of IBD [32]. However, the role of diet may not end at the time of diagnosis of IBD but may also play a role in the disease activity via increasing intestinal permeability, inducing dysbiosis, and stimulating pro-inflammatory processes [33]. Some studies also demonstrated a relationship between dietary components and inflammatory parameters [29,34]. Earlier studies assessing disease activity and UPF intake in adults found a significant association [27,30]. In our cohort, the number of patients with active diseases was low, so we could not reliably analyze the relationship. Interestingly, Vagianos et al. evaluated the association between UPF intake and the number of episodes of active disease and found that active inflammation was significantly greater among participants with UC consuming higher amounts of UPFs. Though they did not find a similar association in patients with CD [30]. In contrast, another study reported that high consumption of UPF was positively associated with clinically active disease (OR = 3.82, 95% CI: 1.49–9.8) in CD [27]. Another study supporting the role of UPF intake in disease progression is the analysis of Chen et al., which shows a clear dose-dependent relationship between UPF intake and colectomy (HR: 4.06, 95% CI: 1.52, 10.86) [28].

If UPF is an important factor triggering the disease activity, then one would expect that those who have diseases that are more difficult to treat have higher UPF intake. However, our data show a different relationship between UPF intake and need for biologicals. A possible explanation for this finding is that children receiving biological therapy—indicative of a more severe or complicated disease course—may be more motivated to engage in lifestyle changes, including dietary modifications, in an effort to support their health and treatment outcomes. This increased adherence to dietary recommendations might reflect a heightened commitment to disease management among those facing greater disease-related challenges.

In our study, we did not find such an association, though there were only a few children with active diseases. Additionally, higher protein and fat intake, as well as reduced fiber intake, in addition to UPF, may be important factors, but these were not included in our analysis [35].

Furthermore, it should be noted that symptoms and mucosal inflammation do not always correlate. In this study, we used disease activity indexes to classify disease activity and found no association between UPF intake and disease activity. However, endoscopic mucosal inflammation or fecal calprotectin are more precise markers of disease activity and may have a relationship with UPF intake.

### 4.1. Strengths and Limitations

A key strength of our study is the use of matched controls and a validated two days of 24 h recall method, which increases the reliability of dietary assessment. Additionally, our stratified analyses provide nuanced insights into the complex relationship between UPF intake and clinical variables such as disease activity, BMI, and treatment modality.

However, some limitations must be acknowledged. The sample size was modest, especially for subgroup analyses, which may have reduced statistical power.

Furthermore, recall bias should be considered in all diet studies, including ours. Secondly, we used the NOVA classification to evaluate UPF intake; however, this method has several limitations, including low interobserver reliability.

### 4.2. Clinical and Research Implications

Nutritional education should emphasize the importance of a diet based on whole foods, while acknowledging the clinical role of certain processed products. Future studies with a larger sample size and prospective design could contribute to the understanding of the relationship between IBD and UPF intake; however, other lifestyle measurements should be assessed in parallel.

## 5. Conclusions

In conclusion, children and adolescents with IBD—especially those with UC—consume more UPFs compared to HC, but this difference is largely influenced by the inclusion of ONS. It is essential to differentiate between clinically indicated UPFs and general dietary patterns to accurately interpret results and develop appropriate nutritional recommendations. According to our data, the UPF intake of children with CD was not lower than HC, suggesting that patients with CD need repeated education to maintain their adherence to the recommended lower intake of UPF.

## Figures and Tables

**Table 1:** Basic characteristics of participants.

	IBD (n = 47)	CD (n = 24)	UC (n = 23)	HC (n = 47)
Age, mean (SD)	15.71 (1.91)	16.11 (1.81)	15.33 (1.96)	15.69 (1.97)
Female, n (%)	20 (42.55)	9 (37.50)	11 (47.83)	20 (42.55)
Age at diagnosis, mean (SD)	12.80 (2.39)	13.39 (1.69)	12.92 (2.69)	-
Disease duration, mean (SD)	2.50 (1.94)	2.50 (1.97)	2.43 (1.91)	-
BMI z-score, mean (SD)	0.12 (1.75)	0.07 (1.96)	0.17 (1.54)	−0.05 (0.82)
PCDAI/PUCAI, mean (SD)	-	3.31 (5.48)	4.78 (9.35)	-
Disease location * for CD or UC (n)				
L1/E1	-	10	5	-
L2/E2	-	5	5	-
L3/E3	-	11	4	-
L4/E4	-	14	7	-
Disease activity				
remission (n)	40	22	18	-
mild (n)	5	2	3	-
moderate (n)	1	0	1	-
severe (n)	0	0	0	-
5-ASA (n)	22	4	18	-
Azathioprine	24	12	12	-
Biologics				
infliximab (n)	7	3	4	-
vedolizumab (n)	2	0	2	-
ustekinumab (n)	1	1	0	-
adalimumab (n)	13	13	0	-

IBD: inflammatory bowel disease, CD: Crohn’s disease, UC: ulcerative colitis, HC: healthy control, SD: standard deviation, BMI: body mass index, PCDAI: pediatric Crohn’s disease activity index, PUCAI: pediatric ulcerative colitis activity index, 5-ASA: 5-aminosalicylates, * disease localization based on the Paris classification.

**Table 2:** Energy and UPF intake in IBD patients based on BMI Z score.

	Underweight (n = 12)	Normal Weight (n = 24)	Overweight (n = 11)	*p* Value
energy intake, mean (SD), kcal	2524 (454)	3073 (1236)	2354 (600)	0.086
UPF, mean (SD), %	48 (15)	50 (15)	53 (13)	0.762

SD: standard deviation, UPF: ultra-processed food.

## Data Availability

The data underlying this article are available in the article and offline. The offline data can be made available upon request from the corresponding author due to limitations in the informed consent obtained from participants, which does not cover open public sharing of raw data.

## Supplementary Materials

The following supporting information can be downloaded at: https://www.mdpi.com/article/10.3390/nu17223532/s1, Supplementary Figure S1. Disease activity and daily ultra-processed food intake as a percentage of total energy intake; Supplementary Figure S2. Daily ultra-processed food intake as a percentage of total energy intake in patients with active disease versus patients in remission for over one year; Supplementary Figure S3. Daily ultra-processed food intake as a percentage of total energy intake in patients with biological therapy versus patients without biological therapy; Supplementary Figure S4. Daily ultra-processed food intake as a percentage of total energy intake in patients with Crohn’s disease and with ulcerative colitis on biological therapy versus without biological therapy; Supplementary Figure S5. Comparing the daily ultra-processed food intake as a percentage of total energy intake in patients with standard biological therapy versus escalated biological therapy.

## Abbreviations

The following abbreviations are used in this manuscript:

BMI	body mass index
CD	Crohn’s disease
CDED	Crohn’s Disease Exclusion Diet
HC	healthy control
IBD	inflammatory bowel disease
MD	mean difference
ONS	oral nutritional support
PCDAI	pediatric Crohn’s disease activity index
PUCAI	pediatric ulcerative colitis activity index
SD	standard deviation
TNF	tumor necrosis factor
UPF	ultra-processed food
UC	ulcerative colitis
