# Supplementary material for: Ultra-Processed Food Intake in Children with Inflammatory Bowel Disease: A Pilot Case–Control Study
Source: Nutrients. 2025 Nov 12;17(22):3532. doi: 10.3390/nu17223532 (PMC12655334; doi:10.3390/nu17223532)
Supplement: Supplementary file 1 [file nutrients-17-03532-s001.zip › nutrients-3892593-supplementary.pdf]

Supplementary material

Ultra-Processed Food Intake in Children with Inflammatory Bowel Disease: A Case-Control Study

Emese Kasznár [1,2], Dorina Bajzát [1,2], Anna Karoliny [1], Judit Szentannay [1], András Szabó [1], Eszter Gombos[1], Vivien Regián[3], Anikó Havasi[4], Erzsébet Pálfi[3], and Katalin Eszter Müller [1,5*]

We categorised food intake according to the NOVA classification system. All food items from the 24-hour recalls were assigned to one of the four NOVA food groups:

NOVA 1: Unprocessed or minimally processed foods that mostly do not contain additional nutrients, such as fruits, vegetables, fresh meat, eggs, milk, grains and legumes.

NOVA 2: Processed culinary ingredients that have undergone minimal processing to make them durable and suitable for seasoning and cooking, as well as ingredients that are not intended for direct consumption, such as oils, butter, salt and sugar.

NOVA 3: Processed foods, which are foods that have undergone preservation processes and are mostly preserved in cans, bottles or packages (e.g. canned tuna, beans, fruit and vegetables; frozen meat and fish).

NOVA 4: Ultra-processed foods (UPFs): foods that are formulations of several food ingredients that include additives for flavour and colour, emulsifiers, and other additives (e.g. sweets, snack products, industrial breads, cereals, margarines, processed meat, and soft drinks).

FIGURES LEGENDS

Supplementary Figure S1. Disease activity and daily ultra-processed food intake as a percentage of total energy intake

Supplementary Figure S2. Daily ultra-processed food intake as a percentage of total energy intake in patients with active disease versus patients in remission for over one year

Supplementary Figure S3. Daily ultra-processed food intake as a percentage of total energy intake in patients with biological therapy versus patients without biological therapy

Supplementary Figure S4. Daily ultra-processed food intake as a percentage of total energy intake in patients with Crohn's disease and with ulcerative colitis on biological therapy versus without biological therapy

Supplementary Figure S5. Comparing the daily ultra-processed food intake as a percentage of total energy intake in patients with standard biological therapy versus escalated biological therapy

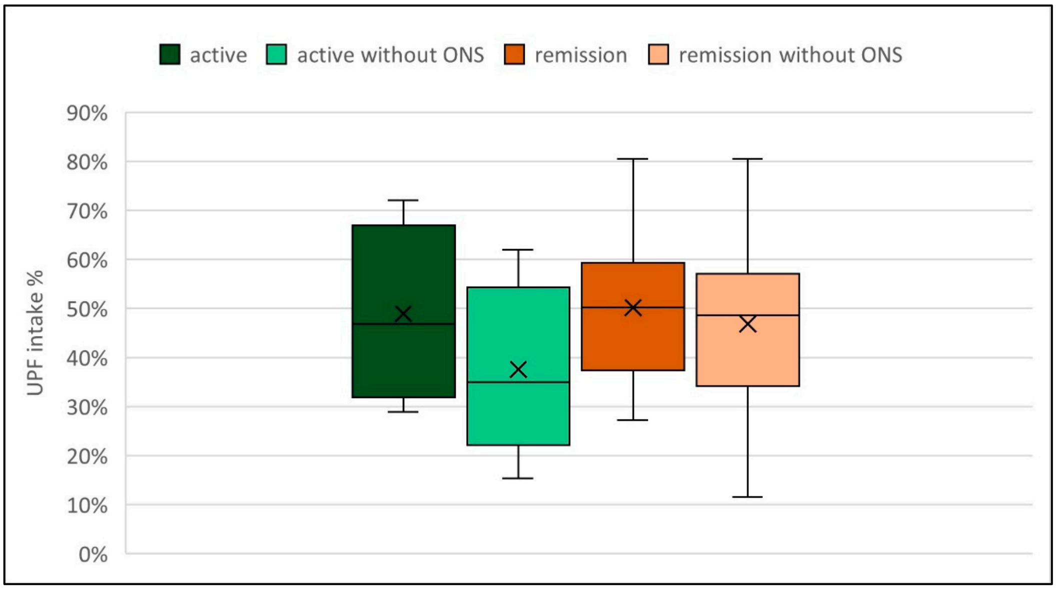

Supplementary Figure S1. Disease activity and daily ultra-processed food intake as a percentage of total energy intake

UPF: ultra-processed food, ONS: oral nutritional supplement

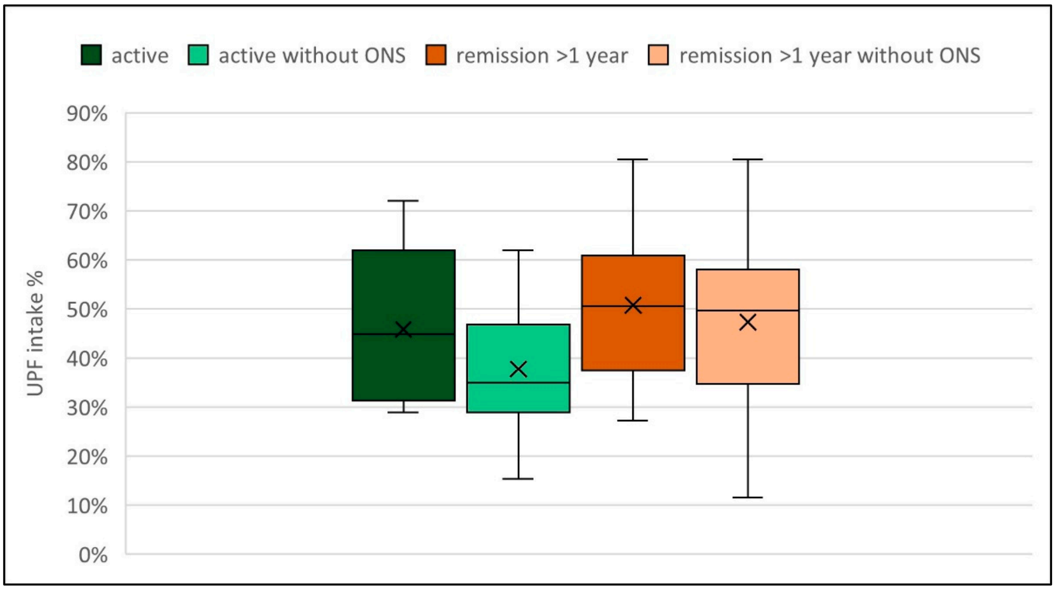

Supplementary Figure S2. Daily ultra-processed food intake as a percentage of total energy intake in patients with active disease versus patients in remission for over one year

UPF: ultra-processed food, ONS: oral nutritional supplement

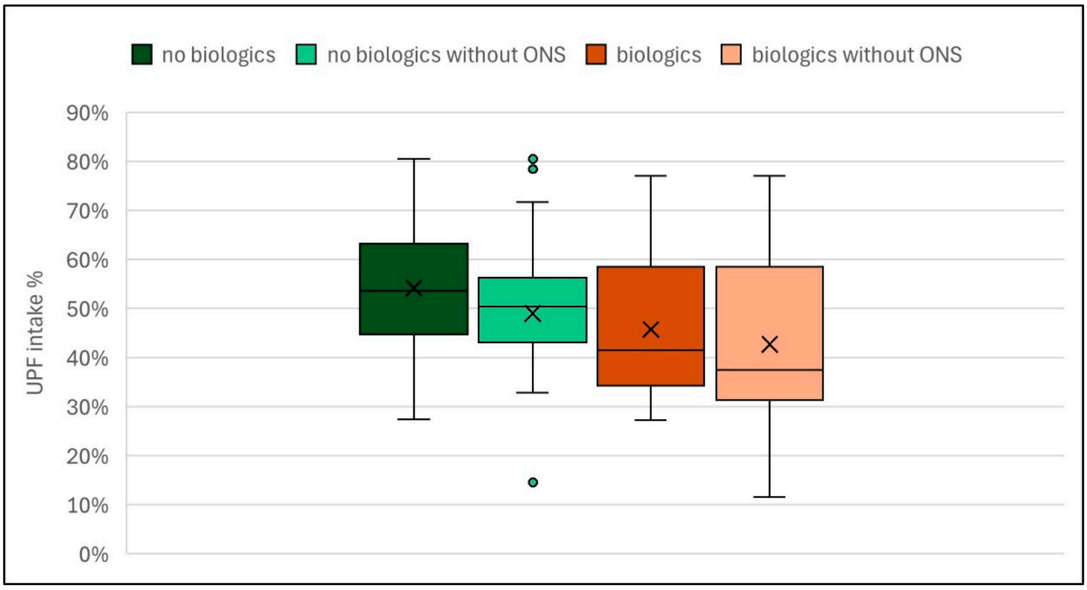

Supplementary Figure S3. Daily ultra-processed food intake as a percentage of total energy intake in patients with biological therapy versus patients without biological therapy
UPF: ultra-processed food, ONS: oral nutritional supplement

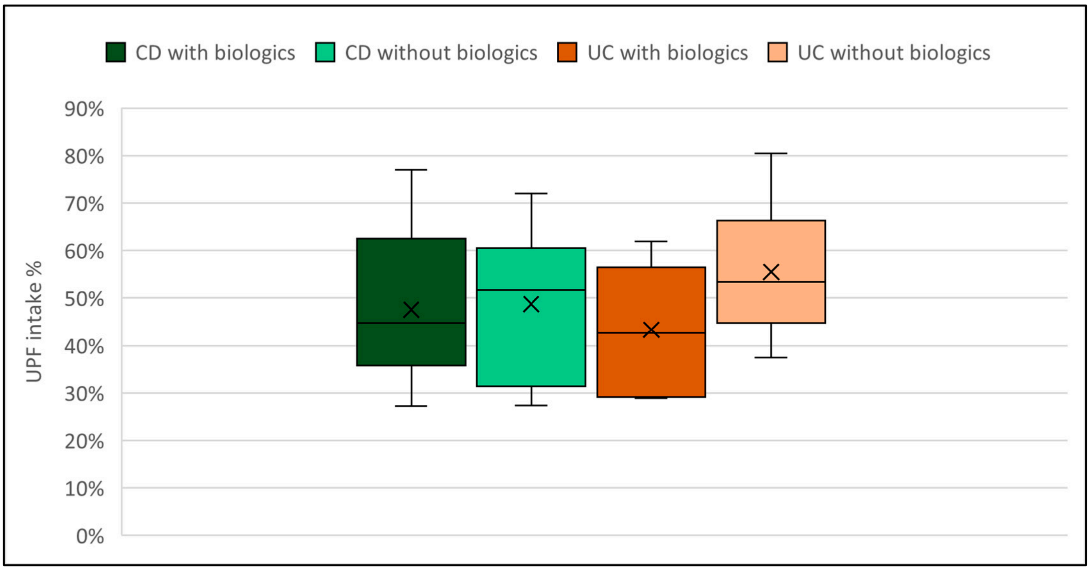

Supplementary Figure S4. Daily ultra-processed food intake as a percentage of total energy intake in patients with Crohn's disease and with ulcerative colitis on biological therapy versus without biological therapy
UPF: ultra-processed food, ONS: oral nutritional supplement, CD: Crohn's disease, UC: ulcerative colitis

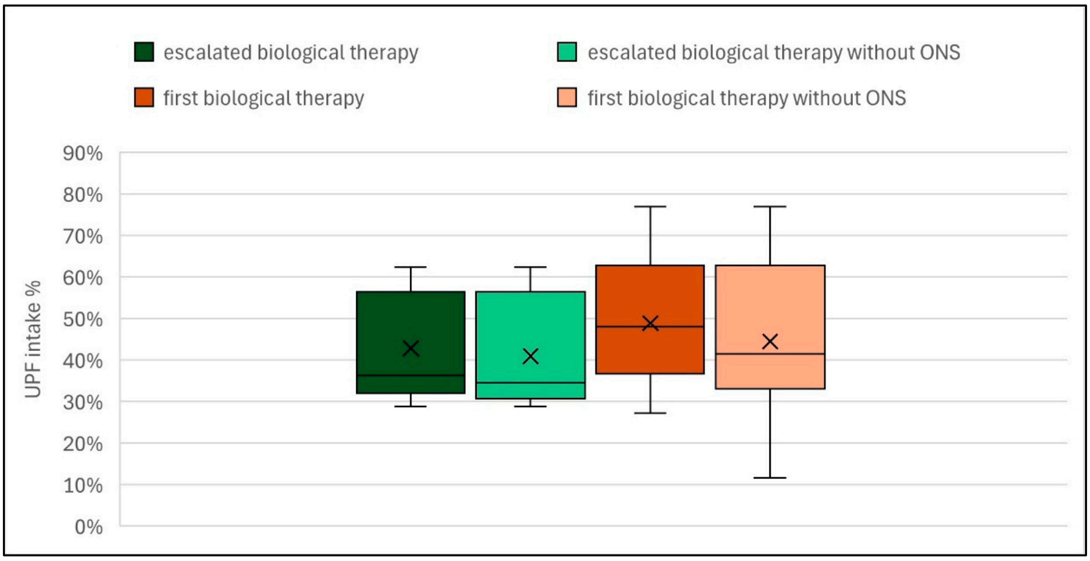

Supplementary Figure S5. Comparing the daily ultra-processed food intake as a percentage of total energy intake in patients with standard biological therapy versus escalated biological therapy
UPF: ultra-processed food, ONS: oral nutritional supplement
